# Bluetongue’s New Frontier—Are Dogs at Risk?

**DOI:** 10.3390/vetsci12050505

**Published:** 2025-05-20

**Authors:** Rita Payan-Carreira, Margarida Simões

**Affiliations:** Comprehensive Health Research Centre (CHRC), Departamento Medicina Veterinária, Escola de Ciências e Tecnologia, Universidade de Évora, 7004-516 Évora, Portugal; mpsimoes@uevora.pt

**Keywords:** BTV epidemiology, BTV pathogenesis, BTV susceptibility, vector-borne diseases, epidemiological surveillance, risk assessment frameworks, host-pathogen/virus interactions, canine abortion, mortality, carnivores

## Abstract

Bluetongue virus (BTV), traditionally considered a pathogen of ruminants, has recently been documented in dogs, challenging conventional understanding of its epidemiology. This narrative review synthesizes emerging evidence regarding BTV infections in domestic and wild carnivores, examining transmission dynamics, pathogenesis, clinical manifestations, and diagnostic challenges. Carnivores can become infected through vector transmission and oral ingestion of infected material. While some infected carnivores remain subclinical, others develop severe clinical manifestations including hemorrhagic syndromes. BTV infection in carnivores is likely underdiagnosed due to limited awareness, nonspecific clinical signs, and absence of established diagnostic protocols for non-ruminant species. The potential role of carnivores in BTV epidemiology remains largely unexplored, raising questions about their function as reservoirs or dead-end hosts. Additionally, carnivores may contribute to alternative transmission pathways and overwintering mechanisms that impact disease ecology. Current biosecurity frameworks and surveillance systems, primarily focused on ruminants, require expansion to incorporate carnivores in viral maintenance and transmission. This review identifies significant knowledge gaps regarding BTV in carnivores and proposes future research directions, including serological surveys, transmission studies, and investigation of viral tropism in carnivore tissues. A comprehensive One Health approach integrating diverse host species, vector ecology, human interference, and environmental factors is crucial for effective BTV control and impact mitigation on human, animals, and environment.

## Simple Summary

Bluetongue, a disease traditionally known to affect sheep and cattle, has recently been found in dogs and other carnivores. This evidence challenges what we thought we knew about how the disease spreads and persists in nature. Our review gathers the latest evidence about bluetongue in carnivores, highlighting that these animals can be infected via insect bites or by eating infected meat. Many cases likely go undetected because veterinarians are not aware of dogs’ susceptibility to this disease. We identify important knowledge gaps and suggest research priorities to better understand the role carnivores play in spreading and maintaining the disease. This information is vital to improve disease control strategies and protect both livestock and companion animals from this emerging threat.

## 1. Introduction

Classically, Bluetongue virus (BTV) is considered a non-contagious, vector-borne pathogen of the *Orbivirus* genus within the *Sedoreoviridae* (formerly *Reoviridae*) family [1]. It is primarily transmitted by *Culicoides* biting midges (Diptera, Ceratopogonidae) and has long been recognized as a significant cause of morbidity and mortality in domestic and wild ruminants [2,3]. As a result, BTV is recognized as an economically important pathogen in the livestock sector [2,4], and is a WOAH notifiable disease.

Reports indicate that BTV has been detected across multiple continents, including Africa, the Middle East, Australia, the South Pacific, the Americas, and Asia. The virus exhibits widespread geographical distribution, with some regions potentially harboring the pathogen without manifesting clinical disease outbreaks [5].

To date, at least thirty-six BTV serotypes have been characterized, some of them labeled as atypical strains [6]. Although BTV serotypes differ in virulence and clinical severity, vascular injury remains a key pathological finding in more aggressive BTV infections. To date, it is accepted that BTV infection via *Culicoides* midges initiates with replication in regional lymph nodes before systemic dissemination through infected mononuclear cells, subsequently spreading to endothelial cells where viral non-structural proteins trigger inflammatory cascades and apoptosis, leading to the observed vascular pathology [7,8]. In livestock species, the virus induces endothelial cell damage that can lead to vascular thrombosis, capillary leakage, thrombocytopenia and coagulopathies, hemorrhage, tissue necrosis, and reproductive complications including abortion, stillbirth, and congenital malformations [9,10]. Mortality rates among susceptible animals exhibit significant variation according to the specific BTV serotype infection [11]. Moreover, the mortality pattern also changes as a result of virus reassortment through time and regions, and possibly co-infections of diverse serotypes [12]. Fulminant BTV conditions also manifest with extensive edema, particularly pulmonary edema, often resulting in animal death, which has been attributed to endothelial dysfunction that increases vascular permeability [13].

While BTV infections have been classically associated with ruminants, emerging evidence indicates that non-ruminant species, namely dogs, are also susceptible to infection. Historically, dogs have not been considered a primary host for BTV, although some initial reports described BTV infection via accidental inoculation with a contaminated live attenuated canine vaccine in USA [14,15,16,17]. However, emerging evidence indicates that canids, both domestic and wild, can become infected [18,19,20,21] through different transmission routes, and it has been suggested that they may interfere with the pattern of the disease dispersion.

BTV spread emerges from the intersection of human activities, climate change, and ecological dynamics [8,22,23]. Livestock movement introduces BTV into new territories, while climate shifts expand Culicoides vector ranges and breeding habitats. Changing temperatures and precipitation patterns alter vector distribution, creating new transmission trends for this virus. Comprehensive surveillance integrating animal movement, vector ecology, and climate data is crucial for predicting and managing BTV transmission.

Recent reports have documented BTV infections in pregnant female dogs in Portugal, particularly in rural areas, shortly after BTV outbreaks in sheep had been reported [24]. Additional sporadic reports appeared in South Africa [20,25] and the Netherlands [26].

Notably, epidemiological data reveal that the majority of naturally occurring BTV infections in dogs disproportionately affect pregnant females and have often a fatal outcome. The vasculotropic and teratogenic properties of BTV could plausibly result in a spectrum of obstetric emergencies in canines, including mid-gestation abortion, dystocia, and maternal hemorrhagic complications, which rapidly deteriorates towards a life-threatening condition due to pulmonary edema and acute renal failure [19,20,25]. This marked reproductive-related state susceptibility suggests a potential tropism of the virus for the pregnant uterus in canines, similar to that observed in other species [20,27], possibly associated with the enhanced vascular proliferation and immunomodulatory characteristic of pregnancy. In infected pregnant dogs, abortion is rapidly followed by death due to acute pulmonary edema [15,17,20,25]. Interestingly, in the study by Hanekom et al. [25], seropositive dogs living on the same farm as the BTV-infected pregnant female and carrying viral RNA in their bloodstream did not present clinical signs of the disease. This apparent susceptibility of pregnant bitches to BTV infection raises significant concerns regarding maternal-fetal health outcomes and highlights the need for targeted surveillance in this vulnerable subpopulation, particularly in regions where BTV occurs and where working and outdoor dogs frequently interact with potentially infected livestock and are exposed to competent vectors.

Despite these potential risks, the veterinary reproductive community remains largely unaware of BTV as a differential diagnosis in canine obstetric complications, even in regions where the virus-induced disease has already been notified. The clinical significance of BTV infection in pregnant dogs remains inadequately characterized despite the growing concerns that reproductive and obstetric complications linked to BTV exposure may be underdiagnosed. This situation presents substantial diagnostic and surveillance challenges, as reproductive failures in dogs are rarely attributed to arboviral infections and BTV testing is seldom incorporated into standard diagnostic protocols for canine obstetric emergencies. Moreover, BTV detection in dogs’ blood [21,25] suggest dogs may play a more significant role in introducing BTV into disease-free areas, and maintain, as reservoirs, the infectious cycle.

Rooted on knowledge acquired in farm animals, this narrative review aims to consolidate current scientific evidence regarding BTV infections in wild carnivores and dogs, with particular emphasis on pregnant bitches experiencing obstetric emergencies or fatal outcomes [20,24]. By critically assessing available data on epidemiology, vector transmission dynamics, clinical presentations, and diagnostic limitations, this analysis highlights key knowledge gaps and proposes future research directions to improve understanding of this overlooked epidemiological aspect of BTV infection. Enhanced surveillance and research into cross-species transmission dynamics are essential, particularly in settings where dogs, livestock, and competent vectors coexist.

## 2. Overview of the Epidemiological Context of Bluetongue Virus

### 2.1. Bluetongue Virus in Brief

Bluetongue virus (BTV) is a complex, non-enveloped double-stranded (ds) virus with a multi-layered structure [28]. Its genome consists of ten dsRNA segments encased in a capsid composed of seven structural proteins (VP1 to VP7) and of non-structural proteins (NS1 to NS4) [28,29,30]. The virus exhibits significant genetic and phenotypic diversity due to rapid evolution through genetic drift, reassortment, and intragenic recombination [29]. The outer capsid protein VP2 is the main determinant of serotype specificity and a target for neutralizing antibodies [31]. Structural differences in VP2’s tip domain between serotypes contribute to antigenic variation [31]. While outer capsid proteins are highly variable, core proteins like VP3 and VP1 are more conserved across the BTV serotypes [29]. This structural and genetic diversity is reflected in the existence of multiple BTV serotypes and impacts vaccine development [31]. These features also allow BTV strains to be classified as eastern and western topotypes, evidencing that BTV isolates from Asia and Africa are considered as genetic origins of European and American isolates [32,33].

BTV, as with other segmented RNA viruses, has the ability to exchange genome segments between different viral serotypes (reassortment), a mechanism that contributes to rapid evolutionary change [34]. Reassortment can drive major adaptive shifts in virus transmission and virulence since it can affect viral replication kinetics, cytopathogenicity, and potentially virulence [35]. The plasticity of BTV allows the virus to reassort not only within field strains [34,36] but also with the live attenuated vaccines serotypes that have been used in Europe [36], raising concerns about the control of the disease.

BTV exhibits a high mutation rate during replication due to the absence of proof-reading mechanisms. This inherent error-prone replication, combined with genome segment reassortment (exchange between co-infecting viral isolates), drives its genetic variability. Of note, reassortment events have been studied in both Culicoides biting-midges and vertebrate hosts [34,37,38]. This mechanism enables novel strain emergence with altered traits like virulence or geographic adaptability [39]. Concurrently, BTV’s vector-borne transmission creates evolutionary pressure to maintain compatibility with both insect vectors and mammalian hosts, limiting genetic changes that could impair replication in either host. This evolutionary flexibility underscores BTV’s capacity to exploit ecological niches and overcome host barriers, posing challenges for outbreak prediction and containment [8].

Most important is the possible co-infection and interplay of diverse BTV serotypes and other biting midge-borne viruses such as the epizootic hemorrhagic disease virus (EHDV, *Orbivirus*) and the Schmallenberg virus (*Orthobunyavirus*). The last-mentioned agent is considered enzootic in Continental Europe, whilst EHDV has only recently been detected in Europe (from 2022 onwards) [40].

### 2.2. Host Range and Species Susceptibility

**Figure 1:**
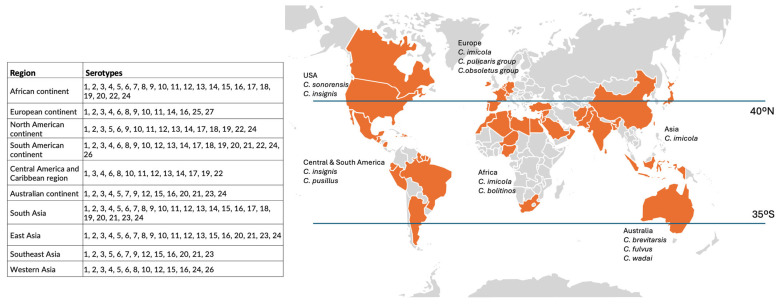
Worldwide distribution of bluetongue disease and representative Culicoides midges species in each region (based on [41]).

Bluetongue viruses circulate in different regions of the world (Figure 1). As of today, BTV has been detected on all continents except Antarctica [10,11]. Over the past decade, the African and the European continents have exhibited the highest BTV serotype diversity. In Asia, BTV-4 and BTV-16 have emerged as predominant serotypes. Meanwhile, in North and South America, bluetongue disease has demonstrated a comparatively lower clinical impact. Australia and Oceania harbor serotypes distinct from those isolated in Europe, with New Zealand maintaining its BTV-free status [41].

The global distribution patterns of BTV serotypes are influenced by the geographic boundaries of different *Culicoides* midge species’ ecosystems (Figure 1), spanning both tropical and temperate climate zones [42], and may colonize new, favorable territories driven by climacteric events such as rare wind-transport events [43]. Over the past decade, the global distribution of bluetongue has expanded dramatically, mirroring trends seen in other vector-borne diseases [44], with numerous outbreaks documented across Europe and Africa. Researchers have identified increased global travel and commerce, widespread deforestation, and accelerating climate changes as primary drivers fueling the vectors’ geographic expansion [22].

Sheep are considered the most clinically susceptible species, frequently developing severe manifestations of disease, whereas cattle and goats often function as subclinical carriers, significantly contributing to viral persistence and maintenance in endemic regions [2,11]. The susceptibility of these species to BTV is influenced by several factors, including genetic predisposition, immune response, and environmental conditions [10].

The shared habitats between domestic and wild species foster cross-species trans-mission and enable the persistence of BTV in diverse ecosystems, contributing to infection reports in some captive or free-living wildlife. Wild ruminants of either the Northern or South Hemisphere regions, including species such as white-tailed deer (*Odocoileus virginianus*), pronghorn antelope (*Antilocapra americana*), and European mouflon (*Ovis aries musimon*), have demonstrated both subclinical infections and fatal disease presentations, depending on the viral serotype and host-related factors [22,45,46,47].

Beyond conventional ruminant hosts, BTV has been sporadically detected in non-ruminant species, such as camelids, both dromedary [48,49] and alpaca [50]. Although antibodies against anti-BTV antibodies have been detected in several carnivore species [14,18,51], only the European lynx [52] and domestic dogs [19,20,24,25] display clinical disease, often fatal. In many cases reported lately, serotype-3 [20,24] has been identified in fulminant spontaneous conditions.

The spectrum of non-ruminant host susceptibility, particularly in species that do not naturally harbor the virus, remains incompletely characterized, raising critical questions regarding cross-species transmission dynamics and their potential epidemiological significance in the global maintenance and dissemination of BTV [42].

Research has highlighted the role of different BTV serotypes in determining the severity of disease across various host species [22,53]. This differential susceptibility underscores the importance of understanding specific interactions between BTV serotypes and host species [54].

### 2.3. Transmission Pathways and Epidemiological Patterns

The transmission of BTV occurs predominantly via hematophagous midges of the genus *Culicoides* [55,56]. Vector competence exhibits substantial geographical variation (Figure 1), with species such as *Culicoides imicola* predominating in Africa and the Mediterranean basin, whereas *C. obsoletus* complex and *C. pulicaris* complex are more prevalent in Northern and Central Europe [23,55]. In North America, the relevant *Culicoides* species is *C. sonorensis* [46,56], while in Central and South America, *Culicoides insignis* and *Culicoides pusillus* have been identified as the main biological vectors for BTV [5]. The climate change-driven alterations in vector distribution have facilitated the expansion of BTV-infection occurrence into previously non-endemic regions, highlighting the dynamic nature of this pathogen’s epidemiology [8,23,57,58,59,60]. Long-term BTV persistence is limited to geographical regions [41] depending on vector overwintering, which is mostly associated with vector survival in particular conditions [9].

Although Culicoides midges are the main vectors for BTV, studies have shown that ticks can become infected with BTV serotype 8, with evidence of transstadial passage in hard ticks and transovarial passage in soft ticks, suggesting they may serve as alternative vectors for the disease [61]. However, additional studies are needed to elucidate the role of ticks in bluetongue epidemiology, particularly regarding their potential as an overwintering mechanism for the virus.

The transmission routes employed by BTV can vary according to serotype classification [7]. While vector-mediated transmission represents the canonical route of infection in ruminants [9], several alternative transmission pathways, vector independent, have been documented, particularly respecting canids and other non-ruminant hosts, underscoring the virus’s remarkable adaptive capabilities. Such alternative infection routes may play a significant role in BTV pathogenesis as mentioned elsewhere.

For domestic dogs, European lynxes, and potentially other carnivores, ingestion of infected placental tissues, aborted fetuses, or contaminated raw meat has been implicated as the putative route in sporadic cases of BTV infection [3,18,24]. This route may be particularly relevant in environments where rural management practices, such as herding dogs feeding on raw placental tissues after calving or lambing events, could inadvertently expose canines to high viral loads, creating a plausible pathway for BTV-associated emergencies. However, this is not the unique route for infection in these animals. Some studies suggest the participation of insect vectors as an alternative infectious route for BTV in dogs [18,19,21,62].

BTV is also capable of crossing the placental barrier in naturally infected pregnant ruminants, potentially leading to abortion, stillbirth, or congenital malformations, particularly some specific serotypes, such as BTV-3 and BTV-8 [63]. This vertical transmission route has significant implications for reproductive management in endemic regions and further potentiates economic losses associated with bluetongue infection of flocks.

Even though bluetongue has been regarded as a “non-contagious” disease, several investigations suggest the possibility of direct transmission between infected and susceptible animals during periods of close contact [64]. Since direct transmission requires close contact of animals, its epidemiologic relevance is often restricted to in-farm or within-group transmission [63]. Similarly, reports of contagion with semen [65,66,67], colostrum [68] serving as viral carriers, and most recently mechanical insect transmission [56] added insightful information on BTV-transmission possibilities. While likely of limited epidemiological significance, transmission via contaminated needles has been postulated [69], along with semen extenders containing contaminated fetal bovine serum [70], and vaccines [14,16], whilst raising the hypothesis that BTV transmission could also occur through other fomites in intensive farming systems [8]. Though definitive evidence on the importance of these non-vectorial routes of infection remains limited, further systematic investigation on epidemiological links is crucial.

Similarly, the relevance of non-vector associated transmission routes for BTV epidemiology and their contribution to pathogenesis and overwintering BTV mechanisms remain poorly quantified. This knowledge gap, combined with insufficient understanding of BTV infection in non-traditional host species, raises an important question that should drive future research, especially as climate change potentially alters vector distributions and abundance.

Since earlier studies, cattle have been claimed as maintenance hosts for certain BTV serotypes and are considered to play an important epidemiological role in disease dispersal. From an epidemiological perspective, due to their propensity to develop prolonged viremia (up to 60–120 days post-infection) without overt clinical signs, cattle may effectively function as reservoirs for subsequent transmission via competent *Culicoides* vectors [7,8,9]. Sheep have also shown viremic stages with mild clinical signs [71], and IFN-mediated immune response in sheep, related to viremia, is considered to be higher and to last longer when compared to cattle [72]. Additionally, other wildlife species, both ruminant and carnivores, have been shown to carry viral RNA in their blood and tissues [73,74,75], contributing to BTV circulation in endemic areas. Currently, long-term BTV persistence is limited to geographic regions characterized by the circulation of two or more virus serotypes [7]. This phenomenon has significant implications for surveillance strategies and control measures in endemic regions.

The complex nature of BTV transmission dynamics demands a comprehensive approach to disease prevention and control [54], addressing multiple transmission pathways simultaneously through strategic vaccination and biosecurity measures to mitigate non-vector-mediated transmission risks. Special attention to vector control is required by means of integrating factors such as environmental conditions, host and vector ecology, vector abundance, immunity and replication events in the vertebrate and invertebrate hosts, BTV-infection status of vectors, anthropogenic driving factors, and viral reassortment and evolution. This data integration could add crucial information for risk assessment and enable better preventive practices (region and species-specific).

### 2.4. Bluetongue in Ruminants and Non-Ruminant Species: Epidemiological Considerations

#### 2.4.1. Disease Expression in Ruminants

Broadly, the severity of the symptoms usually varies with the involved BTV serotype and the host species.

Sheep typically exhibit the most severe clinical manifestations of BTV infection, characterized by fever, oronasal ulcerations, pulmonary edema, and, notably, cyanosis of the tongue and oral mucous membranes (hence the name “bluetongue”) [7,22,76].

Cattle, primarily, develop mild or subclinical disease even when maintaining persistent viremia (often exceeding 60 days post-infection), establishing them as critical epidemiological amplifiers in endemic cycles of certain serotypes [53,54,77]. Nonetheless, recent evidence shows that certain BTV serotypes, particularly BTV-8 or, more recently, BTV-3, can cause reproductive pathologies in cattle, challenging their traditional classification as purely subclinical hosts [53,63,78,79,80].

Goats also typically demonstrate intermediate susceptibility, occasionally developing clinical disease but generally less severe than in sheep, considering the most prevalent circulating serotypes [76,80]. Although their role in the epidemiology of BTV in mixed-farming systems remains incompletely characterized, they might be ancestral hosts for BTV [42]. Certain BTV infections of goat have been characterized as atypical (or goat-associated BTV) [81].

Wild ruminants serve as important sentinel species for BTV circulation in natural ecosystems [46]. Significant mortality events have occurred in various deer populations following the introduction of novel BTV serotypes into naïve populations [47]. These populations can function as maintenance reservoirs [82], sentinel species for (re-)emerging BTV serotypes [45], and potential amplification hosts, particularly at the wildlife-livestock interface [8,47]. Continuous surveillance/monitoring systems should be put in place to study the relevance of co-infections of BTV serotypes/serogroups and/or other *Orbivirus* like Epizootic Hemorrhagic Disease (entailing different host susceptibility and disease severity). Of note, Epizootic hemorrhagic disease virus (EHDV) demonstrates immunological cross-reactivity with BTV isolates. Since 2023, both BTV and EHDV have been co-circulating in Northwestern European countries [83]. Wild species can act as pathogen carriers and potential reservoirs for BTV, with varying susceptibility and clinical manifestations among different species [5]. However, more research is needed to fully understand BTV epidemiology in wild populations.

Since 1998, the European region has reported infections by diverse BTV serotypes, namely BTV-1, 2, 3, 4, 6, 8, 9, 11, and BTV-16. More recently, in September 2023, BTV-3 was laboratory confirmed to be circulating in cattle in The Netherlands [84]. Clinical signs in sheep, cattle, and goats were similar to other BTV serotypes, with no special mention of reproductive issues. Remarkably, goats displayed central nervous system signs [85], while abortion and fatal outcomes in dogs were also recognized [26].

Several reports highlight BTV infection’s role in ruminants’ pregnancy. Significantly associated with the abortive process, congenital infections also relate to neurological malformations, especially for its induced teratogenicity of the central nervous system [86]. Example of these issues are infections in pregnant ewes, which may cause abortion or congenitally malformed lambs, representing a substantial economic burden in endemic regions [4,7]. It is considered that the severity of teratogenic lesions positively relates to earlier gestational period. BTV infections prior to mid-gestation originate congenital BTV infections, with fetuses presenting cavitating central nervous system defects. Descriptions range from severe hydranencephaly to porencephaly (cerebral cysts) [86].

#### 2.4.2. Disease Expression in Dogs and Other Non-Ruminant Hosts

While BTV has traditionally been considered exclusively a pathogen of ruminants, mounting evidence demonstrates that domestic dogs and potentially other carnivores can become infected. Unlike ruminants, the oral ingestion of contaminated ruminant tissues or products was noted as the primary plausible transmission route in carnivores [24,52]. The survival of viable BTV in white-tailed deer carcasses has been demonstrated for 24 h at regular postmortem conditions [87], which can facilitate the virus’s spread to susceptible hosts, impacting livestock health and management strategies. Scavenging species such as wolves (*Canis lupus*), foxes (*Vulpes vulpes*), and other wild carnivores may be at risk due to their opportunistic feeding habits on infected ruminant carcasses. However, new information obtained from spontaneous infection reported in privately-owned domestic dogs with controlled roaming habits suggests that the hematophagous route (vector-borne transmission) is also likely an important route of infection [21], even if definitive proof has not been established in the report by Hanekom et al. [25].

Canine and wild carnivore infections with BTV are increasingly documented both from experimentally induced [14,16,88] and spontaneous cases [20,24,25,52], often presenting with distinct clinical syndromes when compared to ruminants (naturally infected).

A subset of infected dogs developed a severe hemorrhagic disorder, characterized by thrombocytopenia and coagulopathy that caused spontaneous multisystemic bleeding (possibly due to multisystemic vascular injury); petechiae and ecchymoses affecting the skin, gums, and conjunctiva; epistaxis, hematochezia, hematuria, and prolonged bleeding time following minor trauma [16], also manifesting as disseminated hemorrhages in the gastrointestinal tract, lungs, and central nervous system [14]. This syndrome typically develops 5–10 days after consumption of BTV-contaminated ruminant tissues or experimental inoculation, with fatal outcomes in severely affected dogs. These symptoms are not specific to BTV infection, and the differential diagnosis should include other viral hemorrhagic diseases of ruminants that sporadically affect dogs, as well as leptospirosis.

Vasculitis may also be at the origin of reported abortions in pregnant dogs [15], which may later develop fulminant pulmonary edema [19,24,88] or acute renal failure [16]. In a recent study, viral RNA has been identified in uterine and placental samples of dogs infected with BTV-3 (Ct values of 28 and 16, respectively), and its presence in fetal liver and heart (Ct values of 27.57) supports the argument of BTV-3’s ability to cross the placental barrier in these species of carnivores [24].

Although certain BTV serotypes exhibit neurotropism in many species, neurological syndromes were not reported in dogs. However, many infected carnivores exhibit acute respiratory distress syndrome, either alone or in association with the previously described symptoms [21,52], which resembles severe bluetongue cases in ruminants. Affected dogs display tachypnea, dyspnea, and cyanosis, rapidly progressing to respiratory failure as a result of pulmonary edema [15], with serous fluid accumulation in the alveoli and interstitial spaces. Although similar respiratory syndromes may occur in wild carnivores, surveillance data remain limited.

As for the non-domestic ruminants, dogs and wild carnivores may serve as sentinel species for BTV circulation, particularly in regions with limited surveillance in ruminant populations, and enhanced monitoring should be considered. Carnivores are considered dead-end hosts for BTV [89], as the oral infection was considered the most relevant infection route for these species; however, with increasing evidence that the vector-borne and the transplacental transmission routes can also be a possibility, the role of viremic carnivores in BTV epidemiology ought to be carefully evaluated, particularly since BTV-3 isolates from dogs, sheep, and cattle in Portugal were genetically identical [24].

### 2.5. Knowledge Gaps in Carnivores Role in Bluetongue Epidemiology

The epidemiology of BTV infections has been extensively studied in ruminant populations, with considerable attention paid to the virus-vector-host interactions involving *Culicoides* biting midges and domestic and wild ruminants [7,8,90]. However, significant knowledge gaps persist, in particular regarding the role of non-ruminant hosts in BTV epidemiology, concerning carnivores such as domestic dogs and wild carnivore species, as well as the possible role of diverse BTV serotypes co-infection or even other Orbiviruses [91].

Current understanding of BTV epidemiology centers predominantly on the classical transmission cycle involving infected ruminants, competent Culicoides vectors, and susceptible ruminant hosts [11,55,56,92]. This paradigm has shaped surveillance strategies, control measures, and research priorities [93,94]. However, the sporadic detection of BTV in carnivores and the evidence that BTV infection may occur through routes other than vector transmission, even though limited in terms of epizootiology, suggests potential complexity in the transmission dynamics, which remain inadequately characterized.

Whether carnivores could serve as reservoirs capable of introducing BTV into naive ruminant populations has not been adequately investigated. Documentation of natural BTV infection in dogs remains sparse, with limited case reports providing insufficient data to establish incidence rates or susceptibility patterns across different carnivore species [18,20,21,24,25,51,95]. The prevalence of subclinical infections in carnivores is essentially unknown, potentially underestimating the true burden of infection in these populations. The pathogenesis of BTV infection in carnivores has not been systematically investigated, leaving considerable uncertainty regarding species or tissue tropism, viral replication dynamics, and host immune responses in these species.

The clinical manifestation of BTV infection in carnivores presents another substantial knowledge gap. While reported clinical signs have been restricted to pregnant dogs [19,20,24,25,70], the fact that BTV infection may evolve asymptomatically in male and non-pregnant female carnivores [25] hinders the characterization of the spectrum of disease, incubation periods, and serotype-specific pathogenicity differences. This lack of clinical characterization complicates disease recognition and may contribute to underreporting of cases in carnivore populations.

Perhaps most critically, the transmission dynamics involving carnivores remain largely undefined. The fundamental question of whether carnivores function as amplifying hosts capable of contributing to BTV circulation or merely represent dead-end hosts has not been conclusively addressed [21,25,51]. Evidence for direct carnivore-to-carnivore transmission is yet to be proven, and the relative importance of vector-borne versus oral transmission routes in carnivore infection requires clarification, as well as the unveiling on the potential contribution of carnivores to overwintering mechanisms.

Until now, the epidemiological significance of carnivore infections has received minimal research attention. The impact of carnivore infections on BTV maintenance in endemic regions, their potential role in viral persistence during inter-epidemic periods, and their contribution to the geographical spread of BTV, represent substantial knowledge gaps.

In addition, the relationship between *Culicoides* vectors and carnivore hosts constitutes another poorly understood aspect of BTV epidemiology. Though Hanekom et al. [25] were unable to detect dog blood meals in the midges tested, this fact does not exclude the ability of *Culicoides* vectors to transmit BTV to dogs. Data on the feeding preferences of different *Culicoides* species for carnivores in BTV endemic areas and the vector competence for BTV transmission between domestic or wild carnivores and ruminants need to be systematically evaluated.

## 3. Pathogenesis of Bluetongue Infection

The pathophysiology of BTV infection involves complex interactions between virus, vector, and host, with distinct mechanisms for the vector-borne and ingestive routes of infection. The pathophysiological mechanism of the arboviral infection is by far better understood than those associated with other routes of infection. For the purpose of this review, the pathogenesis of the iatrogenic routes of infection will not be mentioned, as they will follow either the vector-borne or one of the non-vectorial pathways.

### 3.1. Vector-Borne Infection

Vector-borne BTV infection is the recognized transmission mechanism for ruminant hosts [52,55]. The pathogenesis of bluetongue involves a complex interplay between virus, vector, and host immune responses, ultimately resulting in a systemic viral infection.

The transmission cycle begins when *Culicoides* midges ingest the virus during blood feeding from an infected host. Initially, the virus targets the vector’s midgut epithelial cells, replicating and subsequently disseminating to secondary organs, with the salivary glands serving as a critical site of viral amplification. Only midges with high viral titers are competent to effectively transmit the virus [57].

Upon inoculation by an infected midge, BTV employs a nuanced primary infection strategy. Local inflammation from vector bites recruits dendritic cells to the site, facilitating additional viral replication [96,97], preceding early immune system recognition steps [98]. The virus first replicates in the skin, with dendritic cells serving as primary targets. These cells then transport the virus to draining lymph nodes [99], establishing the foundation for systemic infection. From the lymph nodes, the virus disseminates to secondary replication sites, including the spleen, lungs, and other lymphoid tissues [100].

BTV targets various cell types, notably the vascular endothelium and mononuclear phagocytic cells (including macrophages, dendritic cells, and lymphocytes) [96,99,101]. This cellular invasion triggers the secretion of proinflammatory and vasoactive mediators, contributing to clinical manifestations such as fever, hyperemia, capillary leakage syndrome, and hemorrhage [99,100,102], as well as the proliferation of BTV-specific CD4+ and CD8+ T cells [99].

The host’s innate immune response plays a critical role in determining the infection’s outcome. Dendritic cells produce interferon 1 (IFN-1), which is crucial for antiviral innate immune responses [8,99,101] and for activating pro-inflammatory cytokine production [98]. At optimal concentrations, interleukins contribute to limiting the early stages of infections and triggering long lasting immune responses. While optimal interleukin concentrations can limit early infection stages and trigger long-lasting immune responses, disruptions in this mechanism can lead to an excessive inflammatory response or “cytokine storm/cytokine hallucination” [8,102], leading to increased vascular permeability, with extensive edema or effusions or even vascular thrombosis.

Notably, BTV has developed mechanisms to circumvent host immune defenses. Despite being traditionally viewed as a potent interferon inducer, the virus can actually impair the canonical IFN-mediated response, thus prompting efficient viral replication [98]. The varying ability of different viral serotypes to bypass immune effects may explain differences in susceptibility across species and variations in clinical severity. Recently, it was demonstrated that the most virulent BTV serotypes/viral isolates show better competence at modulating the IFN response when compared to less virulent serotypes/viral isolates [53,103].

Species-specific variations in BTV infection susceptibility are particularly pronounced. Sheep demonstrate significantly higher disease severity compared to cattle [7,9,99], primarily due to fundamental differences in the reaction time and magnitude of the innate immune response. Higher and earlier IFN-1 concentrations tend to correlate with decreased BTV titers, as observed in cattle compared to sheep, suggesting more efficient early viral replication in sheep cells [104].

The role of specific T-cell subsets in BTV pathogenesis is particularly critical, as the host’s adaptive immune response is primarily mounted through CD8+ T-cell expansion and the production of neutralizing antibodies [8,101]. The immune response depends intricately on the cell types infected and their tissue locations [99]. BTV-infected lymphocytes release increased amounts of IFN-1, triggering apoptosis and subsequent depletion of CD8+ T cells, which contributes to the clinical leukopenia observed in the initial stages of viral replication in ruminant hosts. Notably, the absence of cytotoxic CD8+ T cells correlates with less severe clinical signs [105]. Conversely, the depletion of CD4+ and WC1+ γδ T cells significantly increases disease severity [105]. CD4+ T cells have emerged as pivotal mediators in the immune response. Their absence is associated with impaired humoral immune response as these play a crucial role in facilitating timely neutralizing antibody responses. These cells are particularly important in generating IgG antibodies that target viral non-structural proteins [105].

A unique aspect of BTV pathogenesis, particularly in sheep, is its ability to persistently infect γδ T-cells. This mechanism potentially allows viral overwintering in the absence of vectorial transmission, occurring without clinical signs of viremia, disease, or seroconversion [106]. The interaction between persistently infected γδ T-cells and skin fibroblasts at biting midge feeding sites may enhance virus production, creating an escape mechanism that facilitates continuous Culicoides-mediated transmission [8,106]. Beyond T-cell dynamics interference, BTV infection induces a marked reduction in peripheral B-lymphocytes, further contributing to leukocytosis and the immunosuppressive state in BTV-infected animals [103]. Despite BTV’s capacity to disrupt B-lymphocyte responses and compromise specific antibody production, this function is not completely abrogated; infected animals can still generate antibody responses against BTV, provided their clinical condition is sufficiently favorable [107].

Protective immunity can be achieved through the production of serogroup-specific neutralizing antibodies [108], which forms the basis for vaccination strategies in susceptible farm animals. However, the ability to develop competent humoral immunity varies with BTV serotypes, potentially related to their capacity to impair host IFN-1-mediated response and to disrupt humoral responses [8,107]. In such situations, the protection offered by cellular immune responses alone is generally incomplete [107].

Differences in endothelial cell responses further relate with species resistance to clinical bluetongue conditions. Ovine endothelial cells exhibit reduced prostacyclin release but increased thromboxane production, which enhances coagulation mechanisms and necrosis, contributing to severe pathological manifestations such as pulmonary edema and microvascular thrombosis [8,109].

Ruminants infected with BTV typically exhibit a two-phase viremia pattern, which researchers believe is connected to interferon activity. The initial viremia peak appears to be suppressed by the primary interferon response. However, a subsequent viremia surge often emerges after interferon levels decrease but before adaptive immunity develops sufficiently to eliminate the virus [107]. The detectable duration of viremia varies across different susceptible host species and BTV serotypes. Commonly, sheep experience viraemia lasting between 14 and 54 days, while goats show a similar pattern of viral presence in the blood, ranging from 19 to 54 days. For some serotypes, cattle present a more prolonged viral circulation, with subclinical infections revealing viraemia that can commence as early as 4 days post-infection and potentially persist for 60 to 100 days. This extended viremic period positions cattle as a significant epidemiological reservoir for bluetongue virus transmission [8]. Nevertheless, these arguments are based on old studies, while the detectable viremic period (mammalian hosts) of newly characterized serotypes is lacking research.

Counterarguments are mostly based on studies following outbreaks of the most severe BTV-infections and related impact in farm animals, contributing to a knowledge bias and hampering the full understanding of surviving/convalescent animals and animals without clinical signs to the disease transmission effect [72]. Concurrently, host-immune responses interference to viremic status also require further clarifications as some authors reported IFN modulation on anti-BTV antibodies persistence [8].

### 3.2. Non-Vectorial Route of Infection

The transmission dynamics of BTV extend beyond its canonical vector-mediated route, revealing a complex landscape of viral dissemination that challenges traditional epidemiological understanding.

Beyond the well-established *Culicoides* biting-midge transmission, researchers have identified additional infection pathways, including oral ingestion of contaminated tissues and vertical transmission from infected dams to offspring [63,78,110], which are particularly relevant for carnivore infection. Despite the differences regarding the initial steps of the pathogenic mechanism, those various routes converge in mechanisms common to the one described for the vector-mediated route of infection, demonstrating a level of viral adaptability that extends far beyond traditional vector-borne transmission models.

#### 3.2.1. Transplacental Viral Transmission

The virus’s ability to cross the placental barrier in pregnant infected females represents a critical mechanism of viral spread with significant implications for reproductive health in ruminant populations, whether sheep [111,112], goats [92], or cattle [113,114].

Viral genetic diversity plays a crucial role in BTV transmission and pathogenicity. Different BTV serotypes exhibit marked variability in their capacity for placental invasion and fetal compromise. Besides existing differences in the reported virulence in experimental and spontaneous BTV infections [115], certain related serotypes (e.g., BTV-1, BTV-8, or BTV-3) display pronounced tropism for placental tissues and the fetus [79,86,111], leading to pregnancy losses and severe disruption of fetal development, including congenital malformations and mortality. These virulent serotypes also show enhanced ability to establish persistent infections in fetuses or immunotolerant cows [113,116,117], thus contributing to viral overwintering phenomena in the host [108,112,118].

Despite extensive research, substantial gaps remain in our understanding of BTV’s transplacental mechanisms. Information gathered from experimental models and experimental infections of pregnant females suggests that BTV exploits the unique immunological environment of the placenta to invade fetal tissues [119]. The placental environment provides a unique immunoprivileged site that BTV can exploit: the reduced inflammatory responses, modified immune cell populations, and sophisticated viral immune evasion strategies support the establishment of BTV infection and replication with minimal immune interference. Rojas et al. [120] suggested that BTV may have inherent biological processes that enable placental traversing. Whether these mechanisms involve the existing dendritic cells at the maternal–fetal interface or sialic acid receptors mediated mechanism to enter the cell, as proposed by Hanekom et al. [25], remains to be explored. The placental invasion results in a cascade of pathological processes, including placental inflammation, disruption of placental barrier function, necrosis of the placentome, and impairment of critical nutrients and oxygen transfer mechanisms [119], all of which can compromise fetal development and terminate the pregnancy.

At the level of the fetus, the pathological evidence varies according to the infecting serotype. Van der Sluijs et al. [111] reported the presence of a severe necrotizing encephalopathy and severe meningitis in association with BTV-1 infection in sheep, while BTV-3 infection usually courses with macroscopic fetal hydranencephaly, cerebellar hypoplasia, or porencephaly. In bovines, BTV-8 infection of pregnant females may also trigger fetal death during pregnancy or in the perinatal period, whereas some other fetuses can reach an immunotolerant state and survive [110]. Horizontal transmission via transplacental infection induces viral persistence [63], allowing for a continuous source of infection.

The clinical implications of transplacental BTV transmission are significant and multifaceted, depending on the fetal age at infection [3,86,121]. In ewes and cows, BTV infection during the early stages of pregnancy can have serious neurological consequences for the lambs, while infections during late stage pregnancy may have no apparent effect on the fetus, despite high losses in the first and second thirds of the pregnancy having been reported in some serotype infections [118,121]. In addition to neurological signs, the infected fetus may also display arthrogryposis and muscular atrophy [86].

Still, the immunological mechanisms underlying BTV’s (trans)placental cells invasion remain unclear.

#### 3.2.2. The Mucosal Route of Infection

The direct contact and mucosal routes offer alternative routes of viral entry, circumventing the typical vector-mediated cutaneous inoculation. Exposure to infected bodily fluids—including saliva, colostrum, semen, and blood or even placental tissues — provide the virus with direct access to mucosal epithelial surfaces and underlying lymphoid tissues. This mode of transmission highlights BTV’s remarkable ability to exploit diverse biological interfaces, bypassing initial mucosal barriers and engaging host cellular machinery with remarkable efficiency. The relative importance of these non-vectorial infection routes may differ between the corresponding host species. In ruminant species, saliva, colostrum and semen carrying BTV are unlikely to sustain an epizootic event, although they can contribute to sporadic cases in non-endemic regions. Conversely, the ingestive route of infection may be more relevant for carnivores’ infection. The invading local may determine the entrance pathway used by the virus, but for BTV, this specific pathogenic mechanism remains unexplored.

Infection via seminal contamination, whether in natural breeding or artificial insemination, is of rare occurrence, and has been described for certain BTV-serotypes. Despite the controversial reports, bulls infected with BTV via intrauterine infection may intermittently shed virions throughout their entire life [66], therefore contributing to the venereal contamination of females. The possibility of female infection by artificial insemination was experimentally demonstrated by Bowen and Howard [122], though the authors reasoned that semen contamination with blood might be necessary for successful infection. After introduction into the uterus, the virus pathogenic pathways would follow the mucosal infectious route.

The transmission of BTV through the ingestion of contaminated colostrum has been evidenced, again in a serotype-dependent way. It is possible that blood cells (e.g., lymphocytes) leaking into colostrum or milk in the mammary gland might carry the virus [114]. After ingestion, the virus pathogeny would follow the mucosal infectious route, just like it would happen if the infectious material was BTV-contaminated meat or abortion byproducts. Unlike vector transmission, where the virus is deposited directly into the bloodstream, ingestion of BTV-contaminated material requires survival through the harsh gastric environment. However, the virus may be protected within tissue-bound erythrocytes, which shield viral particles from degradation induced by gastric acid and digestive enzymes [114].

Dendritic cells are key participants of the mucosal immune system [123], where they are present in larger numbers than in the skin. These cells patrol the mucosal environment and contribute to balancing the number of deleterious pathogens by partnering with T-lymphocytes [123]. Thus, it is sensible to anticipate the involvement of dendritic cells in the pathogeny of BTV mucosal route infections.

In the intestine, tissue-resident dendritic cells are relatively abundant, representing about 1% of total cells in the epithelium and lamina propria; they are also found in lymphoid aggregates (GALT—gut-associated lymphoid tissue), bridging the innate and acquire immune response [124,125]. Therefore, entrance in the gut will allow the virus open access to the host. BTV assault on epithelial cells determines a call for additional dendritic cells to the invasion site, initiating the host immune response, as it happens after BTV vectorial inoculation [96,97]. Infected dendritic cells migrate to mesenteric lymph nodes, and from there, BTV proceeds through the systemic lymphatic circulation. From this point on, pathogenesis will possibly mirror vector-borne transmission, with viremia allowing the virus to access secondary replication sites. For the established mucosal infection route, GALT arises as the major early site for viral replication and severe CD4^+^ T-cell depletion. Like other dendritic cells, those in the intestinal tract can produce interferons and other cytokines that may contribute to the virulence of bluetongue.

## 4. Challenges in Diagnosing BTV in Dogs

BTV infections in carnivores are rare and poorly documented, often leading to delayed recognition or misdiagnosis. The nonspecific clinical signs, limited awareness among veterinarians, and absence of established diagnostic protocols for non-ruminant species further complicate timely case identification.

BTV infection in dogs presents variable clinical manifestations, with particularly significant impacts on pregnant females. Initial symptoms include nonspecific signs such as lethargy, weakness, and reduced appetite [20,24]. A particular predisposition exists for BTV to cause disease in pregnant carnivores, with pregnancy complications being prominent, often involving abortion and vaginal discharge. Respiratory symptoms typically progress quickly, characterized by dyspnea, tachypnea, and costoabdominal breathing, accompanied by coarse lung sounds and non-cardiogenic pulmonary edema [20,24]. Systemic complications rapidly develop, including cardiovascular and metabolic disruptions. Clinical manifestations also encompass tachycardia, hypoxia, elevated inflammatory markers, and hepatic dysfunction. Biochemical alterations reveal electrolyte imbalances, acute kidney damage (elevated blood urea and creatinine), and hematological changes, including leukocytosis, anemia, and thrombocytopenia [20]. Gastrointestinal symptoms include vomiting, nausea, and abdominal distension, while systemic decompensation presents as hypothermia and reduced urine production. In severe cases, the disease can lead to euthanasia or mortality despite intensive treatment [20,24].

Importantly, not all BTV-exposed dogs develop clinical symptoms; non-pregnant individuals often exhibit subclinical infections, with pregnancy being a risk factor in disease severity [25]. The complex clinical variability demands comprehensive veterinary evaluation, especially in endemic areas.

The rarity of confirmed BTV infections in dogs and other carnivores significantly impacts disease recognition. Many veterinary laboratories do not routinely test for BTV in carnivores, and the diagnostic protocols for non-ruminant hosts are usually empirical. Several factors further hinder case identification: limited clinical awareness among veterinarians who do not commonly consider BTV as a differential diagnosis in dogs; symptomatic heterogeneity with substantial overlap with numerous more common canine infectious pathologies; and the absence of pathognomonic lesions, as the postmortem findings in infected carnivores are largely nonspecific, further complicating postmortem diagnostics. Consequently, veterinary practitioners must constantly update their differential diagnosis list, integrating the rapidly changing symptomatology, the results of complementary examinations, and the severity of the clinical condition, while remaining open to adopting aggressive therapeutic approaches to minimize further damage and stabilize the patient.

The diagnostic investigation of BTV in symptomatic dogs represents a sophisticated intersection of clinical observation, advanced molecular techniques, and comprehensive epidemiological understanding. Successful diagnosis requires an integrative approach that transcends traditional diagnostic boundaries, embracing the complexity of viral pathogenesis and host-pathogen interactions. Initial clinical evaluation requires a meticulous methodology involving detailed patient anamnesis, comprehensive physical examination, and thorough assessment of clinical manifestations. Veterinarians must critically consider geographical and environmental exposure histories, vaccination records, potential travel-related risk factors, and regional outbreaks status.

The initial indication of a complex clinical presentation is often observed in depressed pregnant females presenting with apparent miscarriage or dystocia, characterized by dark, hemorrhagic vulvar discharge [20,24]. Despite prompt veterinary intervention, including ovariohysterectomy or cesarean section, affected animals typically experience rapid clinical deterioration with progressive multi-organ dysfunction. This poor prognosis and frequent mortality [24] is in blatant contrast to outcomes typically associated with conventional canine obstetric complications. The diagnostic strategy demands a systematic approach to laboratory investigations, encompassing comprehensive hematological, biochemical, and immunological screening to rule out common infectious diseases in dogs, such as leptospirosis, canine herpesvirus, ehrlichiosis, canine distemper, parvovirosis, leishmaniasis, and various rickettsial infections [24].

Hematological investigations should include comprehensive blood count analysis, inflammatory marker assessment, platelet counts, and leukocyte profiling. Biochemical screening protocols must evaluate renal function, hepatic enzyme levels, electrolyte balance, and comprehensive metabolic parameters. A hyperacute rise in creatinine levels paralleling a decrease in the urea-to-creatinine ratio, coexisting with thrombocytopenia and respiratory distress, should raise a red flag.

Special consideration must be given to acute and hyperacute cases of respiratory distress, pulmonary edema, or renal injury that follow pregnancy complications, as these often represent sentinel indicators of potential viral involvement. Comprehensive screening protocols are particularly crucial in endemic regions where vector-borne diseases demonstrate heightened prevalence. The investigative approach should prioritize comprehensive differential diagnosis protocols, maintaining a high index of clinical suspicion while simultaneously remaining receptive to novel diagnostic paradigms.

Diagnosing BTV infection in carnivores raises multiple challenges stemming from low clinical suspicion, nonspecific presentations, and a lack of established diagnostic guidelines. Recognizing the reproductive impacts, the predisposition to renal and pulmonary complications, and the high mortality rate in affected individuals is essential for improving case recognition. Moving forward, targeted research, increased diagnostic surveillance, and interdisciplinary collaboration between companion animals, wildlife, and ruminant veterinarians will be crucial to unraveling the full impact of BTV infection beyond its traditional ruminant hosts.

## 5. Are Biosecurity Risk Assessment Frameworks in Need for Expansion?

Biosecurity, an integrative set of measures to prevent, control, and mitigate risks posed by infectious agents [126], requires adaptation to country-specific contexts while accounting for identified hazards and husbandry particularities. Maclachlan and Mayo [10] emphasize the need for integrated approaches to control Culicoides-transmitted diseases like bluetongue virus (BTV), incorporating vector biology, ecological distribution patterns, and environmental drivers crucial for disease modeling and intervention prediction.

Traditional BTV risk assessments primarily focus on ruminants as recognized viral reservoirs, neglecting the potential role of carnivores despite increasing reports of BTV infections in domestic dogs and other carnivores. This gap is particularly relevant in mixed farming systems and wildlife interfaces where predator-prey dynamics could facilitate virus spillover. Current frameworks fail to adequately account for carnivores in viral maintenance and transmission cycles, representing a critical blind spot in comprehensive BTV epidemiology.

Multiple routes for BTV introduction have been identified, including live animal importation (legal and illegal), natural wildlife migration, germplasm transfer, windborne dispersal of infected vectors, inadvertent vector transport, and administration of inadequately attenuated vaccines [127]. Novel serotype emergence in non-endemic regions is further driven by immunization gaps, insufficient cross-protection, lax biosecurity protocols, climate change-driven vector shifts, and illicit trade networks [44]. The quality of predictive models depends on their ability to integrate these known risk factors, incorporating parameters such as wind patterns for modeling long-distance vector dispersion [128,129] and regional *Orbivirus* incursion magnitude [130].

Current frameworks consider several factors: climatic changes influencing vector dispersal and BTV occurrence in previously low-risk areas [23,127,131,132]; host-pathogen-vector-environment interactions supported by enhanced surveillance; and vaccines offering cross-protection against diverse BTV serotypes [44]. Despite growing evidence, the movement of dogs and wild carnivores from endemic to non-endemic regions remains unrecognized as a risk factor for BTV spread. This is particularly relevant considering the opportunistic scavenging behavior of carnivores, together with the evidence of BTV resilience to environmental conditions [87]. Nonetheless, new studies are required to further investigate viral yields and maintenance in carcasses regarding BTV transmission dynamics and ecological interactions.

Given the mobility of owned dogs (shepherd or companion), stray dogs, and wild carnivores across geographical regions, future risk assessments should account for their potential role in maintaining and disseminating the virus. Integrating carnivores into biosecurity frameworks requires interdisciplinary collaboration between veterinary services, livestock producers, and wildlife conservationists. Surveillance should extend beyond traditional livestock hosts to include domestic and wild carnivores, allowing a more comprehensive understanding of BTV epidemiology.

Preventive measures work most effectively when combined to address diverse aspects of disease transmission. Vector-control interventions should accompany serotype-specific immunization, though implementation methods remain debated. Insecticides used to decrease Culicoides bites include topical treatments, impregnated ear tags, external applications of synthetic pyrethroids or organophosphorus compounds, and indoor spraying [44]. However, concerns exist regarding broad-range insecticides’ impact on biodiversity, particularly pollinators [133].

Biosecurity measures for BTV also include movement restriction and identification of enzootic zones through surveillance and epidemiological investigations. Nelson et al. [130] and EFSA [74] advocate for stringent pre- and post-import testing on ruminants from high-risk areas, quarantine in vector-proof housing, and movement during cold seasons when vector transmission risk is reduced.

The evolving epidemiology of BTV, characterized by reassortment and co-circulation of multiple serotypes, underscores the need for a more comprehensive approach, particularly as climate change reshapes host-vector interactions. While current strategies predominantly target ruminants and vectors, the potential role of carnivores remains underappreciated in transmission ecology. Furthermore, movements of owned dogs from BTV-endemic regions have not been considered as potential risk factors for BTV emergence or as possible contributors to the epidemiological continuum. As evidence consolidates BTV infection in non-ruminants, risk assessment frameworks should incorporate these potential hazards. Beyond laboratory testing, insect repellents should be recommended for dogs to prevent midge bites and possible infection of native vectors. Enhanced measures should include quarantine and testing for dogs from high-risk areas, especially those showing clinical signs or with potential vector exposure.

Combined entomological monitoring, serological surveillance of wild and domestic hosts, and serotype characterization are crucial for effective control programs [44,134]. Strong collaboration between veterinary services, livestock producers, and animal and environmental researchers can sustain adequate surveillance and robust interventions (addressing the identified gaps), reinforcing cross-border responses and supporting One Health-aligned mitigation strategies.

Vaccination strategies for bluetongue are evolving. While inactivated vaccines incur higher costs, they confer sustained immunity without those risks associated with live-attenuated vaccines, such as virulence reversion, genetic reassortment, or teratogenic effects. Emerging platforms, including recombinant vector and subunit technologies, offer advantages including elimination of viral transmission risk, rapid immune responses, and polyvalent formulations for diverse serotypes [44,135,136], especially as cross-protection against co-circulating serotypes are in increasing demand [120].

The World Animal Health Information System (WAHIS) serves as a cornerstone of global animal health governance, with BTV among its listed diseases. WAHIS enhances preparedness through early warning systems, data-driven risk management, and trade regulations, integrating epidemic intelligence tools for proactive threat detection [137]. Biosecurity measures operate within a hierarchical regulatory framework. While certain measures are legally binding, non-compulsory recommendations rely on voluntary stakeholder adherence, influenced by perceived risk and financial feasibility. Resource-intensive protocols, such as including dogs in surveillance systems, may face resistance despite epidemiological benefits, highlighting the need for cost-sharing mechanisms or incentives [126,137].

## 6. Future Directions

While bluetongue virus (BTV) has been extensively studied in ruminants, significant knowledge gaps remain regarding the potential involvement of carnivores in the epidemiology of this disease. Our review highlights several critical areas for future research.

First, systematic serological and virological surveys of domestic and wild carnivores in endemic regions are urgently needed. The occasional detection of BTV antibodies in dogs, cats, and wild carnivores suggests exposure to the virus, but comprehensive studies are lacking. Future investigations should employ standardized methodologies to determine seroprevalence rates across different carnivore species and geographical regions.

The potential role of carnivores as mechanical or biological vectors deserves thorough examination. Carnivores feeding on infected ruminant carcasses may harbor the virus temporarily in their digestive tract or tissues. Investigations on viral persistence in carnivores following consumption of infected material or midges biting and evaluation whether these animals can subsequently further transmit the virus to susceptible hosts or vectors are essential.

Particularly intriguing is the possible contribution of carnivores to overwintering mechanisms. The long-debated “overwintering problem” of BTV might find partial explanation in carnivore involvement. Future studies should investigate whether carnivores can maintain viable virus during vector-free periods and serve as reservoir hosts when vector activity resumes.

Molecular studies examining BTV receptor distribution and cellular tropism in carnivore tissues, especially those at play during carnivore gestation periods, would enhance our understanding of the pathogenesis in these species. While clinical disease appears rare in carnivores, subclinical infections may occur with implications for virus maintenance and evolution.

Additionally, advanced metagenomic approaches could reveal potential adaptations of BTV serotype or strains in carnivore hosts. Viral mutation rates and selection pressures may differ in carnivores compared to ruminants, potentially facilitating host range expansion or altered virulence.

Finally, ecological studies incorporating movement patterns of wild carnivores could elucidate their potential contribution to long-distance BTV spread, complementing our understanding of the virus’s epidemiology beyond vector-mediated transmission.

Addressing these research priorities would significantly advance our comprehension of BTV ecology and potentially inform more effective control strategies for this economically important disease.

## 7. Conclusions

The emerging evidence of Bluetongue virus infections in dogs challenges the long-standing assumption that BTV is strictly limited to ruminants. Although dogs are not primary hosts, their documented infections highlight potential alternative transmission routes and the need for vigilance in disease monitoring. The reviewed literature underscores significant knowledge gaps regarding the clinical spectrum, epidemiological relevance, and risk factors associated with BTV in dogs. Future studies should prioritize understanding the virus-host interaction in carnivores, assess their role in viral maintenance and spread, and explore the potential consequences for disease surveillance.

Veterinary practitioners should remain alert to atypical presentations of known vector-borne diseases in companion animals, particularly in regions with active BTV circulation. The potential implications extend beyond animal health into the realm of public health policy, where accurate risk assessment depends on comprehensive knowledge of disease ecology. Cross-disciplinary collaboration between veterinarians, entomologists, virologists, and epidemiologists will be essential to address these knowledge gaps effectively. As climate change continues to alter vector distribution patterns globally, the scope of BTV surveillance may need to expand to include non-ruminant species. A more comprehensive approach to BTV epidemiology, inclusive of carnivores, will enhance preparedness and response strategies against emerging vector-borne diseases.

## Data Availability

No new data were created or analyzed in this study. Data sharing is not applicable to this article.
